# Fundamental ingredients for discontinuous phase transitions in the inertial majority vote model

**DOI:** 10.1038/s41598-018-27240-4

**Published:** 2018-06-19

**Authors:** Jesus M. Encinas, Pedro E. Harunari, M. M. de Oliveira, Carlos E. Fiore

**Affiliations:** 1 Instituto de Física, Universidade de São Paulo, Caixa Postal 66318 05315-970, São Paulo, São Paulo Brazil; 2 Departamento de Física e Matemática, CAP, Universidade Federal de São João del Rei, Ouro Branco, MG 36420-000 Brazil

## Abstract

Discontinuous transitions have received considerable interest due to the uncovering that many phenomena such as catastrophic changes, epidemic outbreaks and synchronization present a behavior signed by abrupt (macroscopic) changes (instead of smooth ones) as a tuning parameter is changed. However, in different cases there are still scarce microscopic models reproducing such above trademarks. With these ideas in mind, we investigate the key ingredients underpinning the discontinuous transition in one of the simplest systems with up-down *Z*_2_ symmetry recently ascertained in [Phys. Rev. E **95**, 042304 (2017)]. Such system, in the presence of an extra ingredient-the inertia- has its continuous transition being switched to a discontinuous one in complex networks. We scrutinize the role of three central ingredients: inertia, system degree, and the lattice topology. Our analysis has been carried out for regular lattices and random regular networks with different node degrees (interacting neighborhood) through mean-field theory (MFT) treatment and numerical simulations. Our findings reveal that not only the inertia but also the connectivity constitute essential elements for shifting the phase transition. Astoundingly, they also manifest in low-dimensional regular topologies, exposing a scaling behavior entirely different than those from the complex networks case. Therefore, our findings put on firmer bases the essential issues for the manifestation of discontinuous transitions in such relevant class of systems with *Z*_2_ symmetry.

## Introduction

Spontaneous breaking symmetry manifests in a countless sort of systems besides the classical ferromagnetic-paramagnetic phase transition^1,2^. For example, fishes moving in ordered schools, as a strategy of protecting themselves against predators, can suddenly reverse the direction of their motion due to the emergence of some external factor, such as water turbulence, or opacity^3^. Also, some species of Asian fireflies start (at night) emitting unsynchronized flashes of light but, some time later, the whole swarm is flashing in a coherent way^4^. In social systems as well, order-disorder transitions describe the spontaneous formation of a common language, culture or the emergence of consensus^5^.

Systems with *Z*_2_ (“up-down”) symmetry constitute ubiquitous models of spontaneous breaking symmetry, and their phase transitions and universality classes have been an active topic of research during the last decades^1,2,6^. Nonetheless, several transitions between the distinct regimes do not follow smooth behaviors^7–9^, but instead, they manifest through abrupt shifts. These *discontinuous* (nonequilibrium) transitions have received much less attention than the critical transitions and a complete understanding of their essential aspects is still lacking. In some system classes, essential mechanisms for their occurrence^10^, competition with distinct dynamics^11,12^, phenomenological finite-size theory^13^ and others^14–17^ have been pinpointed.

Heuristically, the occurrence of a continuous transition in systems with *Z*_2_ symmetry is described (at a mean field level) by the logistic equation $\frac{d}{dt}m=am-b{m}^{3}$, that exhibit the steady solutions *m* = 0 and $m=\pm \,\sqrt{a/b}$. The first solution is stable for negative values of the tuning parameter *a*, while the second is stable for positive values of *a*. For the description of abrupt shifts, on the other hand, one requires the inclusion of an additional term + *cm*^5^, where *c* > 0 ensures finite values of *m*. In such case, the jump of *m* yields at $a=\frac{{b}^{2}}{4c}$, reading $\pm \sqrt{b\mathrm{/2}c}$. Despite portrayed under the simple above logistic equation, there are scarce (nonequilibrium) *microscopic* models forecasting discontinuous transitions^18^.

Recently, Chen *et al*.^19^ showed that the usual majority vote (MV) model, an emblematic example of nonequilibrium system with *Z*_2_ symmetry^20–22^, exhibits a discontinuous transition in complex networks, provided relevant strengths of inertia (dependence on the local spin) is incorporated in the dynamics. This results in a stark contrast with the original (non-inertial) MV, whose phase transition is second-order, irrespective the lattice topology and neighborhood. The importance of such results is highlighted by the fact that behavioral inertia is an essential characteristic of human being and animal groups. Therefore, inertia can be a significant ingredient triggering abrupt transitions that arise in social systems^5^.

Although inertia plays a central role for changing the nature of the phase transition, their effects allied to other components have not been satisfactorily understood yet^23,24^. More concretely, does the phase transition become discontinuous irrespective of the neighborhood or on the contrary, is it required a minimal neighborhood for (additionally to the inertia) promoting a discontinuous shift? Another important question concerns the topology of the network. Is it a principal ingredient? Do complex and low-dimensional regular structures bring us similar conclusions?

Aimed at addressing questions mentioned above, here we examine separately, the role of three key ingredients: inertia, system degree, and the lattice topology. For instance, we consider regular lattice and random regular (RR) networks for different system degrees through mean-field theory (MFT) treatment and numerical simulations. Our findings point out that a minimal neighborhood is also an essential element for promoting an abrupt transition. Astonishing, a discontinuous transition is also observed in low-dimensional regular structures, whose scaling behavior is wholly different from that presented in complex networks^13^. Therefore, our upshots put on firmer bases the minimum and essential issues for the manifestation of “up-down” discontinuous transitions.

## Model and Results

In the original MV, with probability 1 − *f* each node *i* tends to align itself with its local neighborhood majority and, with complementary probability *f*, the majority rule is not followed. By increasing the misalignment parameter *f*, a continuous order-disorder phase transition takes place, irrespective the lattice topology^20–22^. Chen *et al*.^19^ included in the original model a term proportional to the local spin *σ*_*i*_, with strength *θ*, given by

1 $${w}_{i}(\sigma )=\frac{1}{2}\{1-\mathrm{(1}-2f){\sigma }_{i}\,{\rm{sign}}[\mathrm{(1}-\theta )\sum _{j=1}^{k}{\sigma }_{j}/k+\theta {\sigma }_{i}]\},$$

where sign(*X*) = ±1 and 0, according to *X* > 0, <0 and *X* = 0, respectively. Note that one recovers the original rules as *θ* = 0.

### MFT results

**Figure 1:**
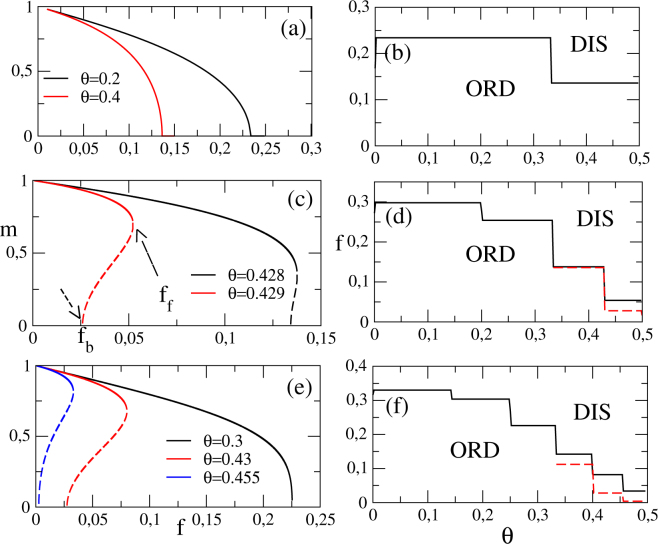
From top to bottom, mean-field results for regular networks for *k* = 4, *k* = 8 and *k* = 12, respectively. The left panels show the behavior of *m* versus *f* for distinct *θ*’s, whereas the right ones show the respective phase diagrams. ORD and DIS correspond to the ordered and disordered phases, respectively. Location of forward and backward transitions are exemplified by arrows in panel (c).

In several cases, a mean field treatment affords a good description of the model properties. By following the main steps from refs^19,21,24,25^, we derive relations for evaluating the order parameter *m* for fixed *f*, *θ* and *k* [see Methods, Eqs (3–8)]. Figure 1 shows the main results for *k* = 4, 8 and 12. Note that MFT predicts a continuous phase transition for *k* = 4 irrespective the value of *θ* [see panels (*a*) and (*b*)], in which *m* is a decreasing monotonic function of the misalignment parameter *f*. An opposite scenario is drawn for *k* = 8 and 12, where phase coexistence stems as *θ* increases [see panels (*c*–*f*)]. They are signed by the presence of a spinodal curve, emerging at *f*_*b*_ [see, e.g., panel (*c*) and (*e*)] and meeting the monotonic decreasing branch at *f*_*f*_. For *k* = 8, the coexistence line arises only when *θ* > 1/3 and is very tiny (*f*_*f*_ − *f*_*b*_ is about 2.10^−4^), but they are more pronounced for *θ* > 3/7. Analogous phase coexistence hallmarks also appear for *k* = 12 (panel (*e*)) and *k* = 20 (Fig. 6 and ref.^19^). Thus, MFT insights us that large *θ* and *k* (*k* ≥ 6) are core ingredients for the appearance of a discontinuous phase transition. A remarkable feature concerning the phase diagrams is the existence of plateaus, in which the transition points present identical values within a range of inertia values. As it will be explained further, that is a consequence of the regular topology. Also, the number of plateaus increase by raising *k*.

### Numerical results

**Figure 2:**
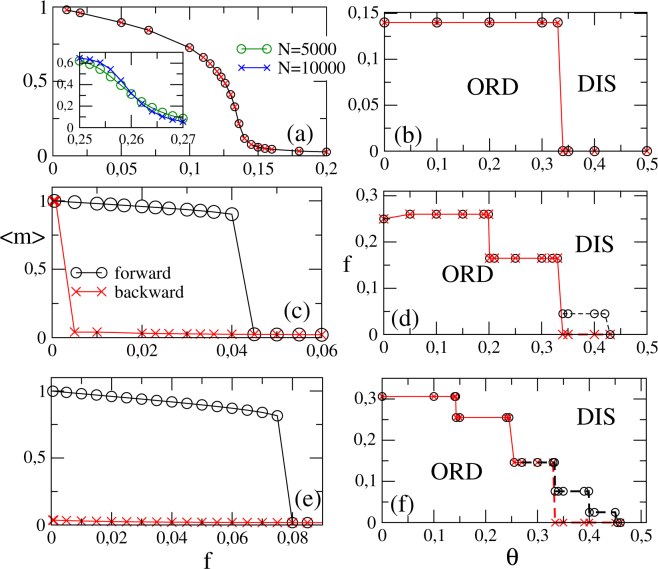
RR Networks: From the top to bottom, numerical results for *k* = 4, *k* = 8 and *k* = 12, respectively. The left panels exemplify the behavior of 〈*m*〉 versus *f* for *θ* = 0.33 (*k* = 4) and 0.35 (*k* = 8 and 12), whereas right ones show the phase diagrams. Inset: Reduced cumulant *U*_4_ vs. *f* for *θ* = 0.05 and *k* = 8. Circles (times) correspond to the increase (decrease) of *f* starting from an ordered (disordered) phase.

Numerical simulations furnish more realistic outcomes than the MFT ones, since the dynamic fluctuations are taken into account. The actual simulational protocol is described in [Methods]. Starting with the random topology, Fig. 2 shows the phase diagrams for *k* = 4, 8, and *k* = 12, respectively.

First of all, we observe that the positions of plateaus are identical than those predicted from the MFT. Also, the phase transition is continuous for *k* = 4, irrespective the inertia value. For *θ* > 1/3, no phase transition is displayed and the system is constrained into the disordered phase. In all cases (see, e.g., Fig. 2(a) for *k* = 8 and *θ* = 0.05), the critical transitions are absent of hysteresis and *U*_4_ curves cross at ${f}_{c}\sim \mathrm{0.261(1)}$ with ${U}_{0}^{\ast }=\mathrm{0.28(1)}$.

Opposite to the low *k*, discontinuous transitions are manifested for *k* = 8 and 12 in the regime of pronounced *θ*. More specifically, the crossovers take place at *θ* = 1/3 and *θ* = 1/4 for the former and latter *k*, respectively. Notwithstanding, there are some differences between approaches. As expected, MFT predicts overestimated transition points than numerical simulations. Although MFT predicts a continuous phase transition in the interval $\frac{1}{4} < \theta  < \frac{1}{3}$ (*k* = 12), numerical simulations suggest that it is actually discontinuous.

**Figure 3:**
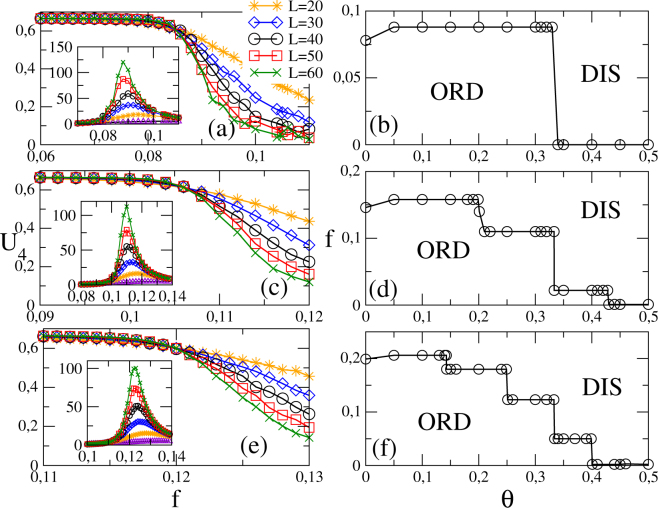
Bidimensional regular lattices for distinct system sizes *N* = *L* × *L*: Left panels show the reduced cumulant *U*_4_ vs *f* for the nearest neighbor (**a**) second-neighbor (**c**) and third-neighbor (**e**) versions, respectively. Inset: the same but for the variance *χ*. Right panels show their correspondent phase diagrams. In all cases, continuous lines correspond to critical phase transitions.

**Figure 4:**
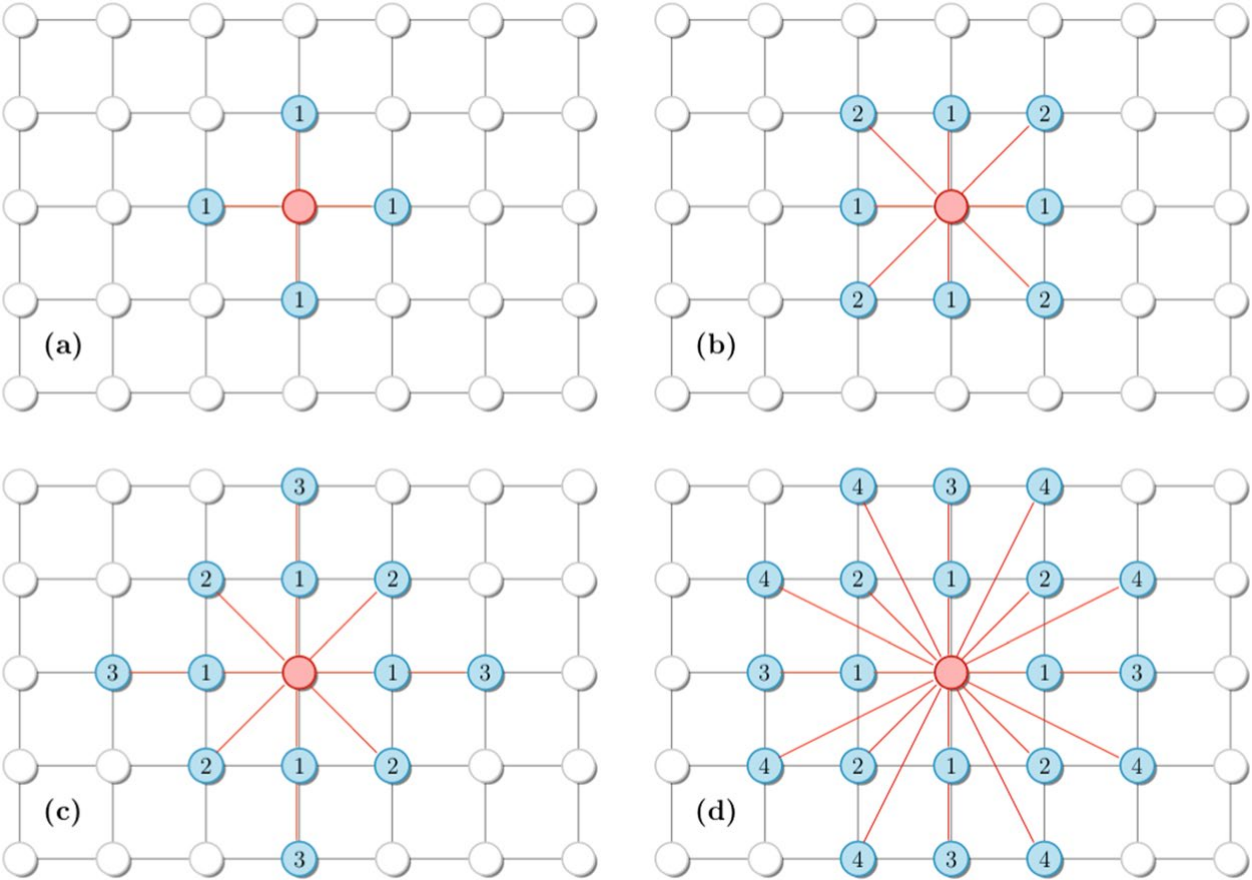
Local configuration for the (bidimensional) versions with interactions between the first (**a**) first and second (**b**) first to third (**c**) and first to fourth (**d**) next neighbors.

In Fig. 3, the previous analysis is extended for regular (bidimensional) versions. In order to mimic the increase of connectivity, the cases *k* = 4, 8 and 12 cases are undertaken by restricting the interaction between the first, first and second, first to third next neighbors, as exemplified in panels (*a*–*c*) in Fig. 4, respectively.

The positions of plateaus are identical than both previous cases, but with lower *f*_*c*_’s. This is roughly understood by recalling that homogeneous complex networks exhibit a mean-field structure, whose correspondent transition points are thus larger than those from regular lattices. Similarly, all critical points are obtained from the crossing among *U*_4_ curves, but the value ${U}_{0}^{\ast }$ is different from the RR case, following to the Ising universality class value ${U}_{0}^{\ast }\sim 0.61$^1,6,20^. Thereby, there is an important difference between random and regular structures: The phase transitions are continuous irrespective the inertia value for *k* from *k* = 4 to *k* = 12.

**Figure 5:**
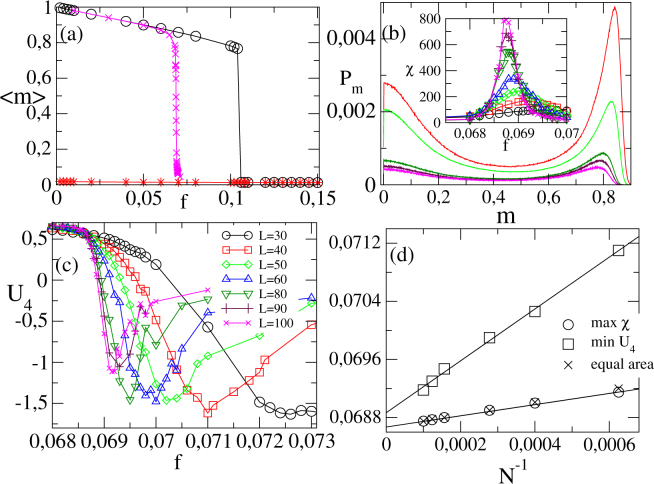
Results for *k* = 20 and *θ* = 0.35: Panel (a) compares the order parameter 〈*m*〉 versus *f* for the RR network (circles and stars) and regular lattice (symbol×) for a system with *N* = 10^4^ sites. Regular lattice case: Panels (b) and (c) show the non normalized equal area probability distribution and the *U*_4_ vs. *f* for distinct *L*’s (*N* = *L* × *L*), respectively. Inset: The variance *χ* versus *f*. In (**d**), the positions of maxima of *χ*, minima of *U*_4_ and equal area versus 1/*N*.

An absolutely different scenario is unveiled by extending interactions range up to the fourth next neighbors spins (mimicking the case *k* = 20) [see, e.g., Fig. 4(d)] and large inertia values, in which the phase transition becomes discontinuous (see, e.g., Fig. 5 for *θ* = 0.35). Contrary to the random complex case, hysteresis is absent [panel (*a*)], on the other hand the order-parameter distribution exhibits a bimodal shape [panel (*b*) shows the value of *f*_*N*_ in which the peaks describing the ordered and disordered phases present equal areas]. Complementary, *U*_4_ behaves quite differently from continuous transitions and presents a minimum whose value decreases with *N* [panel (*c*)]. Also, the maximum of *χ* increases with *N* (inset). These features, analogous than discontinuous absorbing phase transitions^13^, also discloses the emergence of discontinuous transitions in regular lattices as *k* is increased. The *f*_*N*_’s (estimated from the equal area position, maximum of *χ* and minimum of *U*_4_) scales with *N*^−1^ [panel (*d*)], from which one obtains the estimates *f*_0_ = 0.0687(1) (equal area and maximum of *χ*) and *f*_0_ = 0.0689(1) (minimum of *U*_4_) [see Methods for obtaining the finite-size scaling relation].

**Figure 6:**
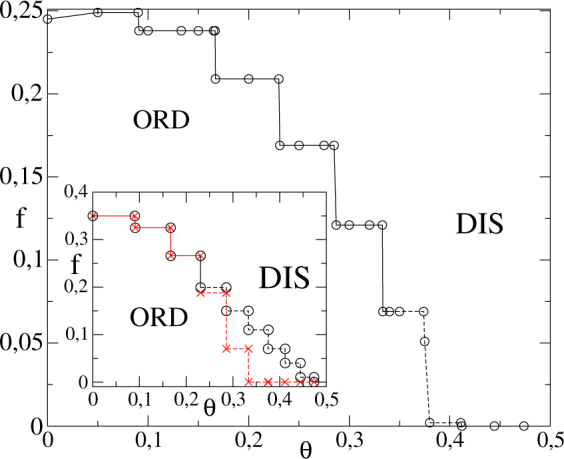
The phase diagram *θ* versus *f* for the MV with *k* = 20 in a bidimensional lattice. Continuous and dashed lines correspond to critical and discontinuous phase transitions, respectively. Inset: The same, but for the RR topology. Circles (×) correspond to the increase (decrease) of *f* starting from an ordered (disordered) phase.

In Fig. 6, the phase diagram is presented. As in previous cases, the positions of the plateaus are identical to the *RR* for *k* = 20 (see inset and ref.^19^). The phase coexistence occurs for *θ* > 1/3, larger than *θ* > 3/13 (RR structure). For *θ* < 1/3, the phase transition is continuous, although *U*_4_ presents a value different from ${U}_{0}^{\ast }\sim 0.61$ in the interval 2/7 < *θ* < 1/3.

## Origin of Plateaus

Since the transition rate depends only on the signal of resulting argument in Eq. (1), the phase diagrams will present plateaus provided the number of neighbors is held fixed. Generically, let us take a lattice of degree *k* with the central site *σ*_0_ with ${n}_{k}^{+}$ and ${n}_{k}^{-}$ nearest neighbors with spins +1 and −1, respectively (obviously ${n}_{k}^{+}+{n}_{k}^{-}=k$). Taking for instance *σ*_0_ = −1 (similar conclusions are earned for *σ*_0_ = 1). In such case, the argument of sign(X) reads $1-\frac{2{n}_{k}^{-}}{k}-2\theta (1-\frac{{n}_{k}^{-}}{k})$, implying that for all $\theta  < {\theta }_{p}=\frac{k-2{n}_{k}^{-}}{\mathrm{2(}k-{n}_{k}^{-})}$ the transition rate −1 → +1 will be performed with the same rate 1 − *f* and thus the transition points are equal. Only for *θ* > *θ*_*p*_ the transition −1 → +1 is performed with probability *f*. Table 1 lists the plateau points *θ*_*p*_ for *k* = 8 and distinct ${n}_{k}^{-}$′s. For example, for ${n}_{k}^{-}=3$ and $0 < \theta  < {\theta }_{p}=\frac{1}{5}$, all transition rates are equal, implying the same *f*_*c*_ for such above set of inertia. For $\theta ={\theta }_{p}=\frac{1}{5}$ the second local configuration becomes different and thereby *f*_*c*_ is different from the value for *θ* < *θ*_*p*_. Keeping so on with other values of ${n}_{k}^{-}$, the next plateau positions are located. It is worth mentioning that ${n}_{k}^{-} > {n}_{k}^{+}$ leads to negative *θ*_*p*_’s, that not have been examined here.

**Table 1:** For the central site *σ*_0_ = −1 and connectivity *k* = 8, the signal function for distinct local configurations.

*σ*_0_	${{\boldsymbol{n}}}_{{\boldsymbol{k}}}^{{\boldsymbol{+}}}$	${{\boldsymbol{n}}}_{{\boldsymbol{k}}}^{{\boldsymbol{-}}}$	X > 0	*θp*
−1	4+	4−	−*θ* > 0	0
−1	5+	3−	$\frac{1-5\theta }{4} > 0$	$\frac{1}{5}$
−1	6+	2−	$\frac{1-3\theta }{2} > 0$	$\frac{1}{3}$
−1	7+	1−	$\frac{3-7\theta }{4} > 0$	$\frac{3}{7}$
−1	8+	0−	1 − 2*θ* > 0	$\frac{1}{2}$

*X* is the value of resulting expression $1-\frac{2{n}_{k}^{-}}{k}-2\theta (1-\frac{{n}_{k}^{-}}{k})$ (see main Text) and *θ*_*p*_ denotes the plateaus positions.

## Methods

We consider a class of systems in which each site *i* can take only two values ±1, according to its “local spin” (opinion) *σ*_*i*_, is “up” or “down”, respectively. The time evolution of the probability *P*(*σ*) associated to a local configuration *σ* ≡ (*σ*_1_, .., *σ*_*i*_, *σ*_*N*_) is ruled by the master equation

2 $$\frac{d}{dt}P(\sigma ,t)=\sum _{i=1}^{N}\{{w}_{i}({\sigma }^{i})P({\sigma }^{i},t)-{w}_{i}(\sigma )P(\sigma ,t)\},$$

where the sum runs over the *N* sites of the system and *σ*^*i*^ ≡ (*σ*_1_, .., −*σ*_*i*_, *σ*_*N*_) differs from *σ* by the local spin of the *i*–th site. From the above, the time evolution of the magnetization of a local site, defined by *m* = 〈*σ*_*i*_〉, is given by

3 $$\frac{d}{dt}m=(1-m){w}_{-1\to 1}-(1+m){w}_{1\to -1},$$

where *w*_−1→1_ and *w*_1→−1_ denote the transition rates to states with opposite spin. In the steady state, one has that

4 $$m=\frac{{w}_{-1\to 1}-{w}_{1\to -1}}{{w}_{-1\to 1}+{w}_{1\to -1}}\mathrm{.}$$

By following the formalism from refs^19,24,25^, the transition rates *w*_−1→1_ and *w*_1→−1_ in Eq. (4) are decomposed as

5 $${w}_{-1\to 1}=\mathrm{(1}-2f){\bar{P}}_{-}+f,$$

and

6 $${w}_{1\to -1}=\mathrm{(1}-f)-\mathrm{(1}-2f){\bar{P}}_{+},$$

where ${\bar{P}}_{-}$ $({\bar{P}}_{+})$ denote the probabilities that the node *i* of degree *k*, with spin *σ*_*i*_ = −1 (*σ*_*i*_ = 1) changes its state according to the majority (minority) rules, respectively. Such probabilities can be written according to

7 $${\bar{P}}_{\pm }=\sum _{n=\lceil {n}_{k}^{\pm }\rceil }^{k}(1-\frac{1}{2}{\delta }_{n,{n}_{k}^{\pm }}){C}_{n}^{k}{p}_{+1}^{n}{p}_{-1}^{k-n},$$

with *p*_±1_ being the probability that a nearest neighbor is ±1 and ${n}_{k}^{-}$ and ${n}_{k}^{+}$ corresponding to the lower limit of the ceiling function, reading ${n}_{k}^{-}=\frac{k}{\mathrm{2(1}-\theta )}$ and ${n}_{k}^{+}=\frac{k\mathrm{(1}-2\theta )}{\mathrm{2(1}-\theta )}$.

Since we are dealing with uncorrelated structures with the same degree *k*, *p*_±_ is simply (1 ± *m*)/2, from which Eq. (4) reads

8 $$\frac{1+m}{2}=\frac{\mathrm{(1}-2f){\bar{P}}_{-}+f}{1+\mathrm{(1}-2f)({\bar{P}}_{-}-{\bar{P}}_{+})},$$

with ${\bar{P}}_{\pm }$ being evaluated from Eq. (7). Thus, the solution(s) of Eq. (8) grant the steady values of *m*.

An alternative way of deriving the MFT expressions consists in writing down the transition rates as the sum of products of the local spins $${w}_{i}(\sigma )=\frac{1}{2}\mathrm{(1}-{\sigma }_{i}{\sum }_{A}{c}_{A}{\sigma }_{A})$$,where *σ*_*A*_ is the product of spins belonging to the cluster of *k* sites, and *c*_*A*_ is a real coefficient. For example, for *k* = 8 and *θ* = 0, we have that $$\frac{d}{dt}m=-\,m+\mathrm{(1}-2f)$$$$\{\frac{35}{16}m-\frac{35}{16}{m}^{3}+\frac{21}{16}{m}^{5}-\frac{5}{16}{m}^{7}\}$$, yielding the critical point $${f}_{c}=\frac{19}{70}$$, in full equivalency with *f*_*c*_ obtained from Eq. (8).

The numerical simulations will be grouped into two parts: Random regular (RR) network and bidimensional lattices.

The former is generated through a configuration model scheme^26^ described as follows: For a system with *N* nodes and connectivity *k*, we first start with a set of *Nk* points, distributed in *N* groups, in which each one contains exactly *k* points. Next, one chooses a random pairing of the points between groups and then creates a network linking the nodes *i* and *j* if there is a pair containing points in the *i*-th and *j*-th sets until *Nk*/2 pairs (links) are obtained. In the case the resulting network configuration presents a loop or duplicate links, the above process is restarted. The bidimensional topologies also present connectivity *k*, but it forms a regular arrangement. Note that both structures are quenched, i.e., they do not change during the simulation of the model.

For a given network topology, and with *N*, *f*, and *θ* held fixed, a site *i* is randomly chosen, and its spin value *σ*_*i*_ is updated (*σ*_*i*_ → −*σ*_*i*_) according to Eq. (1). With complementary probability, the local spin remains unchanged. A Monte Carlo (MC) step corresponds to *N* updating spin trials. After repeating the above dynamics a sufficient number of MC steps (in order of 10^6^ MC steps), the system attains a nonequilibrium steady state. Then, appropriate quantities, including the mean magnetization $\langle m\rangle =\frac{1}{N}\langle |{\sum }_{i\mathrm{=1}}^{N}{\sigma }_{i}|\rangle$, its variance *χ* = *N*[〈*m*^2^〉 − 〈*m*〉^2^] and the fourth-order reduced cumulant ${U}_{4}=1-\frac{\langle {m}^{4}\rangle }{3{\langle {m}^{2}\rangle }^{2}}$ are evaluated, in order to locate the transition point and to classify the phase transition. We have evaluated above averages for a total of 5.10^6^ MC steps.

For both topologies, continuous phase transitions are trademarked by the algebraic behaviors of $\langle m\rangle \sim {N}^{-\beta /\nu }$ and $\chi \sim {N}^{\gamma /\nu }$, where *β*/*ν* and *γ*/*ν* are their associated critical exponents. Another feature of continuous transitions is that *U*_4_, evaluated for distinct *N*'s, intersect at $(f,U)=({f}_{c},{U}_{0}^{\ast })$. Notwithstanding, the set of critical exponents as well as the crossing value ${U}_{0}^{\ast }$ depend on the lattice topology^20,22^. Off the critical point, *U*_4_ reads *U*_4_ → 2/3 and 0 for the ordered and disordered phases, respectively, when *N* → ∞.

In similarity with the MFT, numerical analysis of discontinuous transitions in complex networks is commonly identified through the presence of order parameter hysteresis. For the “forward transition”, simulations are started at *f* = 0 from a full ordered phase (|*m*| = 1), in which the tuning parameter *f* is increased by an amount Δ*f* and the system (final) configuration at *f* used as the initial condition at *f* + Δ*f*. Then, the system will jump to a disordered phase (|*m*| = 0) at a threshold value *f*_*f*_. Conversely, the system evolution now starts in the full disordered phase and one decreases *f* (also by the increment Δ*f*), so that the ordered phase (|*m*| ≠ 0) will be reached at *f*_*b*_. Both “forward” and “backward” curves are not expected to coincide themselves at the phase coexistence. The value of Δ*f* to be considered will depend on *θ*, but in all cases it is constrained between 0.005 and 0.02.

In contrast to complex structures, the behavior of discontinuous transitions is less understood in regular lattices. Recently, a phenomenological finite-size theory for discontinuous absorbing phase transitions was proposed^13^, in which no hysteretic nature is conferred, but instead one observes a scaling with the inverse of the system size *N*^−1^. Here, we extend it for *Z*_2_ up-down phase transitions. Such relation can be understood by assuming that close to the coexistence point, the order-parameter distribution is (nearly) composed of a sum of two independent Gaussians, with each phase *σ* [*σ* = *o* (ordered) and *d* (disordered)] described by its order parameter value *m*_*σ*_ in such a way that

9 $${P}_{N}(m)={P}_{N}^{(o)}(m)+{P}_{N}^{(d)}(m),$$

where each term ${P}_{N}^{(\sigma )}(m)$ reads

10 $${P}_{N}^{(\sigma )}(m)=\frac{\sqrt{N}}{\sqrt{2\pi }}\,\frac{\exp [N\{({\rm{\Delta }}f)m-{(m-{m}_{\sigma })}^{2}\mathrm{/(2}{\chi }_{\sigma })\}]}{[{F}_{o}({\rm{\Delta }}f;N)+{F}_{d}({\rm{\Delta }}f;N)]},$$

where *χ*_*σ*_ is the variance of the *σ*–gaussian distribution, Δ*f* = *f*_*N*_ − *f*_0_ denotes the “distance” to the coexistence point *f*_0_ and each normalization factor *F*_*o*(*d*)_ reads

11 $${F}_{o(d)}({\rm{\Delta }}f;N)=\sqrt{{\chi }_{o(d)}}\,\exp \{N{\rm{\Delta }}f[{m}_{o(d)}+\frac{{\chi }_{o(d)}}{2}{\rm{\Delta }}f]\}\mathrm{.}$$

Note that (10) leads to the probability distribution being a sum of two Dirac delta functions centered at *m* = *m*_*o*_ and *m* = *m*_*d*_ at *f* = *f*_0_ for *N* → ∞. For *f* − *f*_0_ → 0_+(−)_, one has a single Dirac delta peak at *m* = *m*_*d*_ (*m*_*o*_ ≠ 0). The pseudo-transition points can be estimated under different ways, such as the value of *f*_*N*_ in which both phases present equal weight (areas). In such case, from Eq. (10) it follows that ${P}_{N}^{(o)}(m)={P}_{N}^{(d)}(m)$ for

12 $$({f}_{N}-{f}_{0})\,[({m}_{o}-{m}_{d})+\frac{({\chi }_{o}-{\chi }_{d})}{2}({f}_{N}-{f}_{0})]=\frac{\mathrm{ln}\,[{\chi }_{d}/{\chi }_{o}]}{2}\frac{1}{N}\mathrm{.}$$

Since *N* is supposed to be large, the right side of Eq. (12) becomes small and thus (*f*_*N*_ − *f*_0_) is also small. By neglecting terms of superior order (*f*_*N*_ − *f*_0_)^2^, we have that

13 $${f}_{N}\approx {f}_{0}+\frac{\mathrm{ln}\,[{\chi }_{d}/{\chi }_{o}]}{\mathrm{2(}{m}_{o}-{m}_{d})}\frac{1}{N},$$

implying that the difference *f*_*N*_ − *f*_0_ scales with the inverse of the system size *N*. Evaluation of the position of peak of variance *χ* provides the same dependence on *N*^−1^, whose slope is the same that Eq. (13) [see, e.g., panel (*d*) in Fig. 5].

In this work, we have classified the (discontinuous) phase transition in regular lattices based on the behavior of the three aforementioned quantities.

## Conclusions

A discontinuous phase transition in the standard majority vote model has been recently discovered in the presence of an extra ingredient: the inertia. Results for distinct network topologies revealed the robustness of such phase coexistence trademarked by hysteresis, bimodal probability distribution and others features^19^. Here, we advanced by tackling the essential ingredients for its occurrence. A central conclusion has been ascertained: discontinuous transitions in the MV also manifest in low dimensional regular topologies. Also, its finite size behavior (entirely different from the network cases), is identical to that exhibited by discontinuous phase transitions into absorbing states^13^. This suggests the existence of a common and general behavior for first-order transitions in regular structures. In addition, low connectivity leads to the suppression of the phase coexistence, insighting us that not only the inertia is a central ingredient, but also the connectivity. Numerical simulations reveal that for random regular networks, the minimum neighborhood is *k* = 7, whereas about *k* = 20 is required for changing the order of the transition in bidimensional lattices. Summing up, the present contribution aimed not only stemming the key ingredients for the emergence of discontinuous transitions in an arbitrary structure, but also put on firmer basis their scaling behavior in regular topologies.
